# In‐Memory Mathematical Operations with Spin‐Orbit Torque Devices

**DOI:** 10.1002/advs.202202478

**Published:** 2022-07-10

**Authors:** Ruofan Li, Min Song, Zhe Guo, Shihao Li, Wei Duan, Shuai Zhang, Yufeng Tian, Zhenjiang Chen, Yi Bao, Jinsong Cui, Yan Xu, Yaoyuan Wang, Wei Tong, Zhe Yuan, Yan Cui, Li Xi, Dan Feng, Xiaofei Yang, Xuecheng Zou, Jeongmin Hong, Long You

**Affiliations:** ^1^ School of Optical and Electronic Information & Wuhan National Laboratory for Optoelectronics Huazhong University of Science and Technology Wuhan 430074 China; ^2^ Faculty of Physics and Electronic Science Hubei University Wuhan 430062 China; ^3^ School of Physics Shandong University Jinan 250100 China; ^4^ School of Computer Science and Technology & Wuhan National Laboratory for Optoelectronics Huazhong University of Science and Technology Wuhan 430074 China; ^5^ Department of Physics Beijing Normal University Beijing 100875 China; ^6^ Institute of Microelectronics University of Chinese Academy of Sciences Beijing 100029 China; ^7^ School of Physical Science and Technology Lanzhou University Lanzhou 730000 China; ^8^ Shenzhen Huazhong University of Science and Technology Research Institute Shenzhen 518000 China; ^9^ Wuhan National High Magnetic Field Center Huazhong University of Science and Technology Wuhan 430074 China

**Keywords:** analog mathematical computing, image and signal processing, in‐memory computing, neural network, spin‐orbit torque

## Abstract

Analog arithmetic operations are the most fundamental mathematical operations used in image and signal processing as well as artificial intelligence (AI).  In‐memory computing (IMC) offers a high performance and energy‐efficient computing paradigm. To date, in‐memory analog arithmetic operations with emerging nonvolatile devices are usually implemented using discrete components, which limits the scalability and blocks large scale integration. Here, a prototypical implementation of in‐memory analog arithmetic operations (summation, subtraction and multiplication) is experimentally demonstrated, based on in‐memory electrical current sensing units using spin‐orbit torque (SOT) devices. The proposed structures for analog arithmetic operations are smaller than the state‐of‐the‐art complementary metal oxide semiconductor (CMOS) counterparts by several orders of magnitude. Moreover, data to be processed and computing results can be locally stored, or the analog computing can be done in the nonvolatile SOT devices, which are exploited to experimentally implement the image edge detection and signal amplitude modulation with a simple structure. Furthermore, an artificial neural network (ANN) with SOT devices based synapses is constructed to realize pattern recognition with high accuracy of ≈95%.

## Graphical abstract

This work reports a prototypical implementation of in‐memory analog mathematical operations based on spin‐orbit torque devices. Several orders smaller area overhead and comparable power consumption are obtained compared to the complementary metal oxide semiconductor counterparts, but the data can be locally stored in the configuration. Edge detection and amplitude modulation are experimentally demonstrated while pattern recognition is simulated.

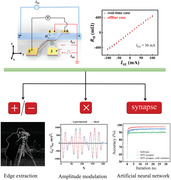

## 1 Introduction

A major criticality of current digital computing based on complementary metal oxide semiconductor (CMOS) transistors, is related to the required computational area/power, which does not scale well with the problem complexity.^[^ ^1^ ^]^ In addition, the scaling trend of CMOS performance has slowed down because of the power wall and slower voltage scaling.^[^ ^2^, ^3^ ^]^ Moreover, the constant data shuttling between the information processing and memory units in the traditional von‐Neumann architecture also significantly limits the speed, area, and energy efficiency.^[^ ^3^, ^4^ ^]^ Along with the unprecedented development of artificial intelligence (AI) and the Internet of Things (IoT), an exponential growth in the amount of data requires new insight into high area/energy efficiency and powerful computing paradigm.^[^ ^5^ ^]^ Many works have focused on beyond CMOS devices and beyond von‐Neumann architectures like in‐memory computing (IMC) based on nonvolatile memory (NVM) that executes computing tasks directly within the memory array.^[^ ^6^, ^7^, ^8^, ^9^, ^10^ ^]^ Meanwhile, under error‐tolerant circumstance, compared to the digital processor, analog computing presents a promising and possibly revolutionary paradigm in recent years, as it does not need analog‐to‐digital conversion and can allow massively parallel operations.^[^ ^11^, ^12^, ^13^ ^]^ Therefore, it is crucial to explore in‐memory analog computing (IMAC), offering an attractive solution to the energy consumption and area issues.

The key challenge of IMC is to realize it without impacting the desirability of the resulting design as a standard memory.^[^ ^14^ ^]^ Due to these constraints, conventional IMC based on NVM is typically limited to perform simple specific operations, for example, currently focusing on the parallel multiply‐accumulate (MAC) operations, which are the primary calculations used in AI, with a crossbar memristors array network.^[^ ^15^, ^16^, ^17^ ^]^ Specifically, the current at each cross point is the product of input voltage and memristive conductance which can be modulated in an analog manner and are widely used as synaptic weight in neuromorphic computing, following Ohm's law for multiplication, and the total current at each column is a summation of the current at each cross point according to Kirchhoff's current law.^[^ ^18^ ^]^ However, in this IMC scheme, it is impossible to realize analog arithmetic multiplication of the same kind of signals and real‐time storing of the computational results, which limits the application scenarios of IMAC. In addition, these memristive devices suffer from deficiencies, including nonlinear and asymmetric weight‐update characteristics with an additional reset operation, constraining the performance of artificial neural networks (ANNs) for neuromorphic computing.^[^ ^19^, ^20^ ^]^

Here, we introduce spin‐orbit torque (SOT) devices to experimentally realize in‐memory analog mathematical operations such as summation, subtraction, and four‐quadrant multiplication, to implement the general purpose applications such as image or signal processing for edge computing. The possible circuits composed of discrete components, for performing such analog arithmetic operations, have been designed in the past based on memristor.^[^ ^21^, ^22^ ^]^ But these proposals have suffered from substantial limitations, including relatively large size and slow response. Our main idea is to exploit the SOT devices with perpendicular magnetic anisotropy (PMA) to linearly sense and store (in‐memory sense) the electrical currents as anomalous Hall resistance (AHR). In addition to nonvolatility and scalability, the CMOS‐compatible SOT technique further possesses low energy consumption, high speed and endurance. Thus, SOT devices offer an avenue for dense IMAC paradigms. The summation or subtraction of the currents, is realized by Kirchhoff's law, and thus, an in‐memory of analog summation/subtraction in the SOT devices is configured. If the other input current (reading current) is applied into the SOT device, its anomalous Hall voltage would be proportional to the multiplication of the reading current and sensed current which is proportional to AHR, thereby implementing the analog four‐quadrant multipliers. Meanwhile, the SOT devices whose AHR is linearly changed with sensed current, can be used as artificial synapses to construct an ANN with initialization‐free MAC operations for pattern recognition.

## 2 Results and Discussion

### 2.1 In‐Memory Electrical Current Sensing Unit

**Figure 1:**
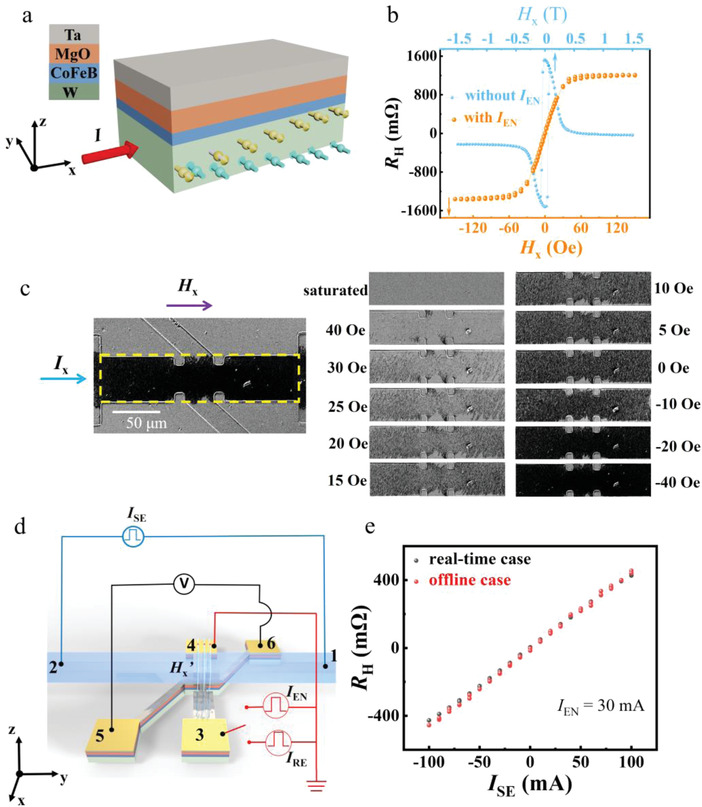
Basic in‐memory sensing unit. a) Schematic of the SOT device with a stack consisting of W(5)/CoFeB (1.1)/MgO (2)/Ta (2) (from the substrate side, unit is nm). b) *R*_H_ as a function of an external in‐plane field (*H*_x_) with and without a collinear enable current *I*_EN_. c) MOKE images captured after the application of *I*_x_ together with *H*_x_. Note that the device is saturated by +200 Oe magnetic field first. d) Schematic of the basic in‐memory electrical current sensing unit and circuits for measurements. *I*_SE_ represents the current flowing in the sensed current path and *H*_x_’ denotes the generated in‐plane field in the SOT device by *I*_SE_. *I*_RE_ is the reading current for offline sense case. e) *R*_H_ as a function of *I*_SE_ for the real‐time (black dots) and offline (red dots) sensing cases.

The SOT heterostructure device (W/CoFeB/MgO/Ta from the bottom, **Figure** **1**a) shows hysteresis of anomalous Hall effect (AHE) loop with sharp switching, indicating a strong PMA (Section S1, Supporting Information). The magnetization can be switched by an in‐plane current *I*_x_ with the assistance of a collinear magnetic field *H_x_* via SOT (Section S1, Supporting Information). Moreover, as found in our previous work,^[^ ^23^ ^]^ the coercive field of the AHE loop (AHR *R*_H_ vs *H_x_*) decreases with *I_x_* increases. Here, at *I_x_*  = 30 mA, *R*_H_ varies linearly with applied *H_x_* within the range of −12 to +12 Oe (orange points in Figure 1b). It means the SOT device can sense the magnetic field along *x* direction, under the assistance of *I_x_* (named enable current *I*_EN_, *I*_EN_ is 30 mA/0.5 s unless otherwise specified). In contrast, without *I*_EN_, a more than 3 kOe in‐plane field is needed to drive the demagnetization and thus changes *R*_H_ to ≈0 (blue points in Figure 1b). The mechanism of magnetic field sensing can be understood that the *I*_EN_ causes the demagnetization state of device, and thus a domain nucleation‐dominated magnetization reversal is very sensitive to the collinear magnetic field, either direction or magnitude, caused by SOT.^[^ ^24^, ^25^ ^]^ It was proved by the magneto‐optical Kerr effect (MOKE) microscopy investigation. The MOKE images (Figure 1c) depict that the proportion of −*M*_z_ domains (shown in black) grows in a dispersed manner when scanning *H*_x_ from +40 to −40 Oe, together with *I*_x_ = 30 mA, after initializing the magnetization to the saturated state under +200 Oe. The nonvolatile variation of magnetic domain structure makes the SOT device feasible to memorize the sensed magnetic field. Correspondingly, the SOT device is possible to sense and memorize the electrical current as *R*_H_ of the heterostructure, via the magnetic field generated by itself. Indeed, in our basic in‐memory electrical current sensing unit (here named SOT unit), the SOT device with a current path (consisting of a 60 nm thick gold metal track) on the top surface separated by 50 nm thick Al_2_O_3_ insulating layer, *R*_H_ varies linearly with the sensed current *I*_SE_ (flowing in the Au path) scanning backward and forward from +100 to −100 mA, under the *I*_EN_. It is noted that *I*_SE_ only generates a magnetic field along the *x* direction (*H*_x_′) (Section S2, Supporting Information), with the corresponding range of −12 to +12 Oe in the heterostructure (Section S3, Supporting Information). On the contrary, without the *I*_EN,_ the in‐plane magnetic field generated by the sensed current is too small to affect the *R*_H_ as discussed above. Thus, when *I*_EN_ is switched on, *I*_SE_ can be real‐time sensed and stored as *R*_H_ in the heterostructure. Otherwise, the heterostructure offline stores *I*_SE_ at the moment when *I*_EN_ is switched off and the memorized *R*_H_ can be readout by applying a small reading current (named *I*_RE_, *I*_RE_ is 0.1 mA 0.1/s unless otherwise noted). Figure 1e shows the *R*_H_ versus *I*_SE_ curves with a linear relationship for the real‐time (black dots) and offline (red dots) cases, respectively. Therefore, *R*_H_ can be expressed as *R*_H_ = *k* × *I*_SE_ for both cases, where *k* is the slope of the *R*_H_–*I*_SE_ curve by linear fitting, if we neglect the AHR offset mainly resulting from contact misalignment and sample inhomogeneity.^[^ ^26^ ^]^ Noting that, in general, in the real‐time sensing case, *k* is lower than that in the offline case for the same *I*_EN_, due to the Joule heating effect caused by lasting relatively high current *I*_EN_ in the former case, for example, *k* is 4.6 Ω A^−1^ (offline) and 4.4 Ω A^−1^ (real‐time) under *I*_EN_ = 30 mA (Section S4, Supporting Information). As expected, with increasing *I*_EN_, the *k* difference would become more distinct (Section S4, Supporting Information).

### 2.2 In‐Memory Analog Summation/Subtraction and Edge Detection

**Figure 2:**
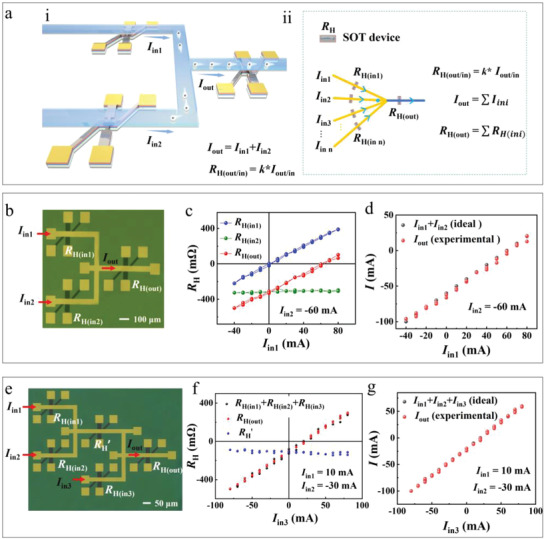
In memory analog summation/subtraction based on interconnected SOT units. a(i)) Schematic of the interconnected network with three basic units for implementing in‐memory analog summation, where the sensed current paths of the three units are connected to a node. (ii) Schematic of summation computing architecture with much more connected units. b) Optical microscopy image of the connected three units following the architecture described in the schematic. c) *R*_H_ as a function of input current *I*_in1_ for the individual SOT devices in the three units (*R*_H(in1)_, *R*_H(in2),_ and *R*_H(out)_). d) The comparison between the ideal *I*_out_ (i.e., *I*_in1_ + *I*_in2_) values (black dots) and the *I*_out_ (red dots) extracted from the measured *R*_H(out)_ through the relationship *I* = *R*_H_/*k*, for the network with three connected units. e) Optical microscopy image of the network with three input currents. f) In this network, the summation of collected *R*_H_ (*R*_H(in1)_, *R*_H(in2)_, and *R*_H(in3)_) as a function of *I*_in3_ (black dots). The red and blue dots denote the collected *R*_H(out)_ and *R*_H_’. g) The comparison between the ideal *I*_out_ (*I*_in1_ + *I*_in2_ + *I*_in3_) values (black dots) and the extracted *I*_out_ (red dots) for the network with three inputs.

Many SOT units can be correlated to perform useful functions by building an interconnected network where their sensed current paths are connected to a node. Following this architecture, a simple configuration for connecting three SOT units is schematically shown in **Figure** **2**a(i). The electrical currents in the sensed current paths meeting at the common node have to satisfy Kirchhoff's current law stating the sum of the currents flowing into the node (Input current *I*_in(1,2)_) is equal to the sum of the currents flowing out of the node (output current *I*_out_), *I*_out_ = *I*_in1_ + *I*_in2_, thereby realizing analog current summation function. Of course, the current subtraction operation can be also easily obtained as *I*_in1(2)_ = *I*_out_ − *I*_in2(1)_. Meanwhile, the branch (input/output) electrical current in the network can be sensed and memorized by the corresponding SOT device (as *R*_H_) under the branch current path at each SOT unit, that is, *R*_H(in/out)_ = *k* × *I*_(in/out)_. These indicate one SOT‐device's *R*_H_ can tell us not only the value of its corresponding branch current but also the calculation result of other two branch currents, if we know their operation relationship among the currents, for example, *R*_H(out)_ = *k* × *I*_out_ = *k* × (*I*_in1_+*I*_in2_). If we detect every individual SOT‐device's *R*_H_, we can immediately know every branch current and also their operation relationship. These are also true for much more connected SOT units (Figure 2a(ii)). Therefore, in‐memory analog summation/subtraction can be performed in such interconnected SOT units.

Figure 2b shows the three fabricated identical SOT units following the above interconnected architecture. In this configuration, we applied simultaneously two pulse currents with 0.5 s pulse duration serving as input currents, which are on the left side of the node, and then the *R*_H_ (*R*_H(in1)_, *R*_H(in2)_, and *R*_H(out)_) were measured after *I*_EN_ is switched off (offline sensing case). Here, *I*_in1_ scans forward and backward between 80 and −40 mA with a step of 10 mA, while *I*_in2_ remains constant of −60 mA. As we found in the independent SOT unit, *R*_H(in1)_ is proportional to its corresponding *I*_in1_ and the proportional coefficient *k* is ≈5 Ω A^−1^ (Figure 2c). It is also observed that, the curve of *R*_H(out)_ as a function of *I*_in1_ is nearly parallel to the curve of *R*_H(in1)_ versus *I*_in1_ with an offset equal to *R*_H(in2),_ indicating that the three *R*_H_ values manifest a perfect summation relationship, *R*_H(out)_ = *R*_H(in1)_ + *R*_H(in2)_. This summation relationship also holds for different *I*_in2_ values (for example, 0, −20 and −40 mA), indicating the stability of the scheme (Section S5, Supporting Information). On the other hand, once we know the *k*, the summation of two input currents, i.e., *I*_out_, can be derived from *R*_H(out)_ measurement. The obtained *I*_out_ using this scheme are in good agreement with the theoretical calculated value of the two input currents summation (Figure 2d). For further investigating the extendibility of the scheme, we fabricated an interconnected network with three input currents (*I*_in1,2,3_) (Figure 2e). The *I*_in1_ and *I*_in2_ remain constant (*I*_in1_ = 10 mA, *I*_in2_ = −30 mA), while *I*_in3_ is scanned between +80 and −80 mA. From the measurement results, the *R*_H(out)_ is equal to the summation of three independently collected *R*_H_ values (*R*_H(in1)_, *R*_H(in2)_, and *R*_H(in3)_), which corresponds to those input currents (Figure 2f). Similarly, the *I*_out_ obtained from *R*_H(out)_ coincides well with the theoretical summation of three input currents (Figure 2g). It is worthwhile to mention that the intermediate *R*_H_’, monitoring output of *I*_in1_ and *I*_in2,_ remains nearly constant and equals to the intercept of *R*_H(out)_ versus *I*_in3_ curve, i.e., *R*_H(in1)_+*R*_H(in2)_. The same behaviors are observed for different combinations of (*I*_in1_, *I*_in2_), including (20, 30 mA), (−10, 30 mA), (10, −10 mA), and (−20, −30 mA) (Section S6, Supporting Information). These results indicate our in‐memory analog computation scheme can be extended for much more inputs.

**Figure 3:**
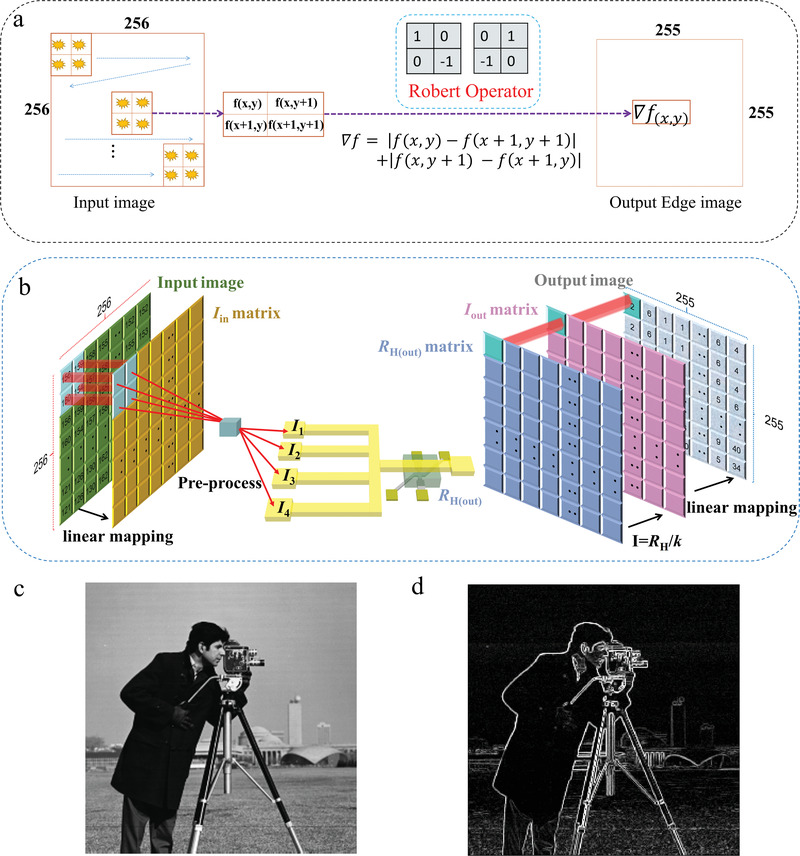
Edge detection by the in‐memory analog summation/subtraction computing. a) The principle of obtaining the edge pixels in a 2D image by Robert operator. 𝑓(*x*,*y*) denotes the grayscale value at location (*x*,*y*) in the original image while 𝛻𝑓(*x*,*y*) represents the obtained gradient magnitude. b) Schematic of the process to realize the edge detection by our adder/subtractor. c) The original image and d) Output image after edge detection. “cameraman” image reproduced with permission from MIT under a Creative Commons Attribution Non‐Commerical license (https://creativecommons.org/licenses/by‐nc/4.0/).

Then, we exploit our adder/subtractor to experimentally realize edge detection, one of the fundamental operations in image processing, for an original 8‐bit grayscale image with 256 × 256 pixels. Gradient‐based Robert operator is used to find edge pixels (or detect edge lines) in the image (**Figure** **3**a). The approximate gradient magnitude (𝛻𝑓) based on the Robert operator is obtained by computing the summation of the absolute values of the differences between diagonally adjacent pixels in the surrounding 2 × 2 grayscale matrix, that is, 𝛻𝑓 = |𝑓(𝑥,𝑦)−𝑓(𝑥+1,𝑦+1)| + | 𝑓(𝑥,𝑦+1)−𝑓(𝑥+1,𝑦)|, where 𝑓(𝑥,𝑦) denotes the grayscale value at location (x,y) (Section S7, Supporting Information). To obtain the gradient magnitude by the current analog computation scheme, the grayscale values of the original image (Figure 3c) are linearly mapped to current values from 0 to 50 mA as inputs of the adder/subtractor. For simplicity, we assigned positive (negative) signs to the larger (smaller) values in the diagonal positions for each 2 × 2 input current matrix. By applying the processed 2 × 2 current matrix to a four‐input computing configuration, the gradient magnitude in current form is sensed and memorized in the output SOT device as *R*_H(out)_ (Figure 3b). We sequentially processed this image from the top‐left to the bottom‐right corner. As a result, an *R*_H(out)_ matrix and thus the corresponding current matrix with dimensions of 255 × 255 were obtained. The edge extraction image obtained by remapping the current to a grayscale matrix is shown in Figure 3d. We investigated the pixel differences between the ideal output and the output from our scheme. The output obtained with our analog adder/subtractor computations scheme closely matches the ideal output, with a standard deviation of 2.95% in the pixel values (Section S8, Supporting Information). The small deviation is mainly due to the nonideal linearity between *R*_H_ and *I*_SE_.

### 2.3 In‐Memory Analog Multiplication and Amplitude Modulation

**Figure 4:**
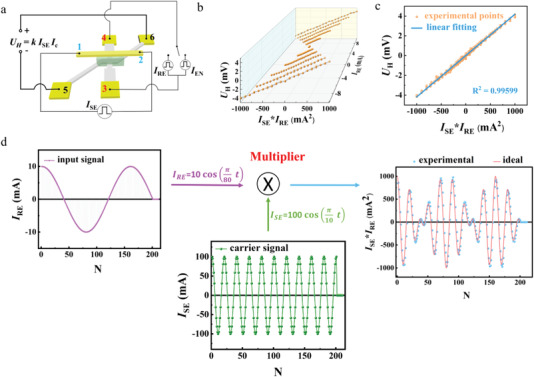
In‐memory multiplication based on a basic unit. a) Schematic of the four‐quadrant analog multiplication scheme. b) At a given constant *I*_RE_, *U*_H_ is plotted as a function of *I*_SE_ × *I*_RE_ with *I*_SE_ changes in a range of ±100 mA with a step of 10 mA, when *I*_RE_ varies from −10 to 10 mA with a step of 2 mA. c) Linear fitting between *U*_H_ and *I*_SE_ × *I*_RE_ to all observed data shown in b) with an R‐square of 0.99 599. d) Implementation of amplitude modulation utilizing in‐memory four‐quadrant analog multiplication.

For a single SOT unit, the anomalous Hall voltage *U*_H_ is expressed as *U*_H_ = *R*_H_ × *I*_c_, where *I*_c_ is the current applied along terminals 3 and 4 (**Figure** **4**a). Additionally, as established above, *R*_H_ can represent the current *I*_SE_ flowing in the sensed current path, *R*_H_ = *k* × *I*_SE_, as *I*_SE_ varies between −100 and 100 mA. Therefore, *U*_H_ can be defined as an analog product of the two current signals *I*_SE_ and *I*_c_, *U*_H_ = *k* × *I*_SE_ × *I*_c_, if *k* is a constant and independent of *I*_SE_ and *I*_c_. Corresponding to real‐time and offline current‐sensing case, *I*_c_ is *I*_EN_ and *I*_RE_, respectively. In the real‐time case, *k* slightly varies with applied *I*_EN_ ranging from 30 to 37 mA, but the variation is insignificant (less than 5%) in our measurements (Section S4, Supporting Information). Thus, this approach can still be used for analog multiplication in cases that allow a small reduction in precision. On the contrary, in the offline case, it is found *k* can remain constant for *I*_RE_ ranging from −10 to +10 mA (Section S9, Supporting Information). We plot *U*_H_ as a function of *I*_SE_ × *I*_RE_, while both *I*_RE_ and *I*_SE_ are variable. At a given *I*_RE_, one straight line was obtained, when *I*_SE_ was scanned between −100 and 100 mA with the 10 mA step, denoting *U*_H_ is proportional to the product of *I*_SE_ and *I*_RE_. With the different *I*_RE_ varying from −10 to 10 mA with 2 mA step, a group of parallel straight lines were obtained. The negligible deviations from a linear fitting between *U*_H_ and *I*_SE_ × *I*_RE_ to the entire measurement data confirm that the proportional coefficient *k* is indeed a constant (Figure 4c).

Once we know the *k*, the product of any *I*_SE_ and *I*_RE_ ranging within [−100 mA, +100 mA] and [−10 mA, +10 mA], respectively, can be easily achieved from the detection of *U*_H_. Therefore, in‐memory four‐quadrant analog multiplier is realized in a single SOT unit. The four‐quadrant analog multiplier is an important building block for many analog signal processing applications, such as filters, modulators, and mixers.^[^ ^27^, ^28^ ^]^ Here, we experimentally implement amplitude modulation (AM), which is a widely used modulating form in wireless communications, by our multiplier where the input signal is used as *I*_RE_ and the carrier signal without modulation is considered as *I*_SE_ (Figure 4d). In our experiment, the amplitude variations of both the input (*I*_RE_) and carrier (*I*_SE_) signals follow cosine waveforms, whose expression are $10\cos(\frac{\pi }{80}\;t)$ and $\;100\cos(\frac{\pi }{10}\;t)$, respectively. Upon imposing *I*_RE_ and *I*_SE_ on the SOT unit, the analog multiplication of these two current signal is directly obtained by measured *U*_H_ divided by *k* (*U*_H_/*k*), which displays a signal whose amplitude is modulated by the input signal but whose frequency is consistent with that of the carrier signal. In addition, the experimental results agree well with the calculated products of *I*_SE_ and *I*_RE_ ($1000\cos\left(\frac{\pi }{10}\;t\right)\cos\left(\frac{\pi }{80}\;t\right)$).

### 2.4 ANN Simulations for Handwritten Digit Recognition

Since the SOT device can sense *I*_SE_ as the *R*_H_ form, the *R*_H_ of the SOT device is possible to be continuously modulated by *I*_SE_. Therefore, we can consider the SOT devices as artificial synapses (named SOT synapses), whose weights are tuned by *I*_SE_ flowing in the Au layer of the SOT unit, to construct an ANN for IMC. We simulated the ANN with experimentally measured characteristics of the SOT synapse to perform image recognition using the MNIST (Modified National Institute of Standards and Technology) database of handwritten digits, whose image size is 28 × 28.

**Figure 5:**
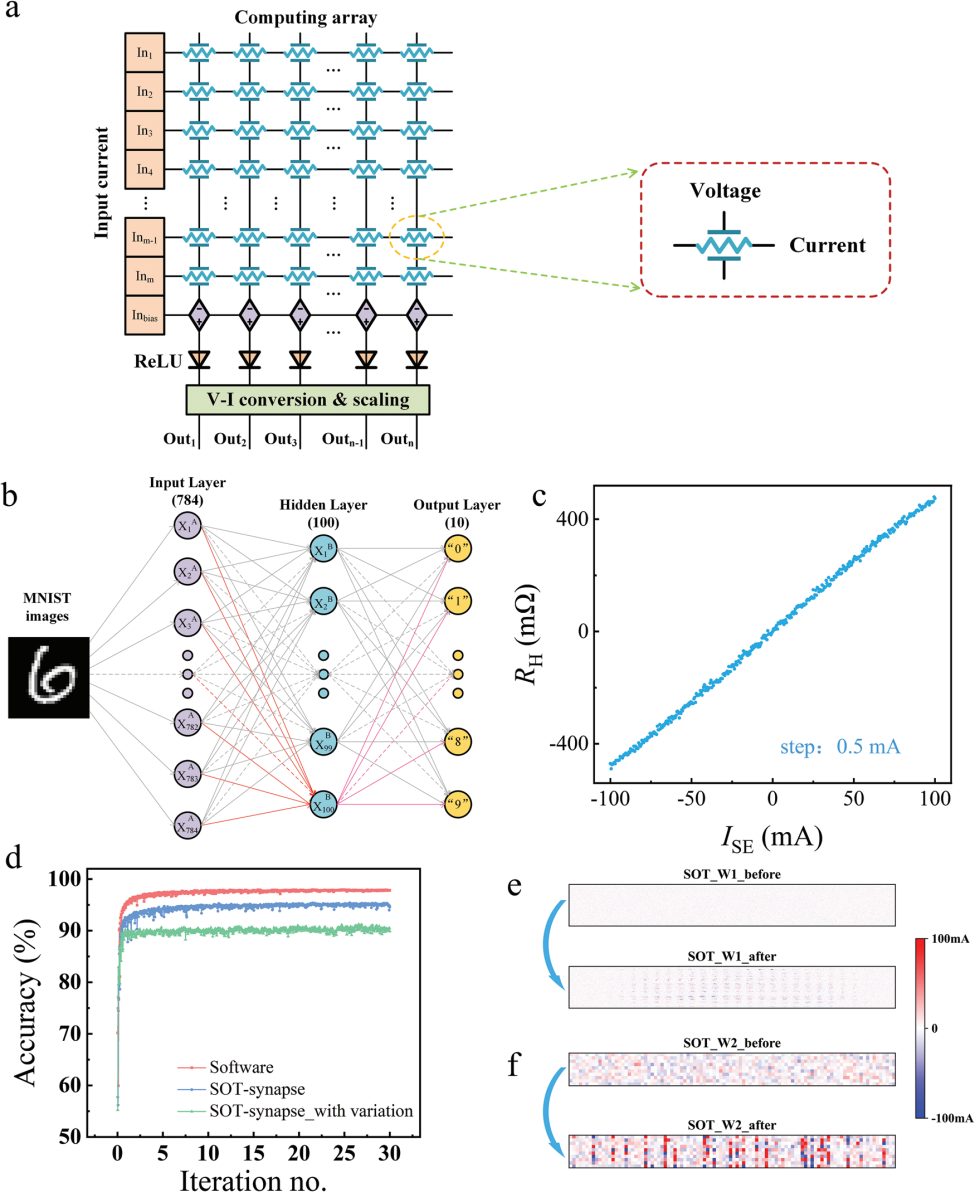
Fully connected neural network simulations for handwritten digit recognition based on MAC operations. a) MAC operations implemented in a crossbar array based on AHE structures. b) The fully‐connected neural network structure consists of 784 input neurons, 100 hidden neurons, and 10 output neurons. c) Experimentally measured *R*_H_‐*I*_SE_ data points of an SOT‐based synapse with a step of 0.5 mA. d) Pattern recognition accuracy as a function of training iteration where a batch size of 300 images is used. Defect‐free SOT‐based synaptic devices reduce accuracy slightly (blue line), comparing to the software‐based ones (red line). Moreover, the variation in SOT‐based synaptic devices will degrade accuracy (green line). e) Evolution of the SOT synapse weights of input‐hidden synaptic matrix before and after in situ training. f) Evolution of the SOT synapse weights of hidden‐output synaptic matrix before and after in situ training.

The crossbar array based on AHE structures of SOT synapse is illustrated in **Figure** **5**a. At each cross point, the *R*_H_ of the SOT device is locally stored as a synaptic weight and *U*_H_ is the product of *I*_RE_ (serving as inputs of the ANN) and *R*_H_, as discussed above. In each column, the Hall voltage detection terminals are connected in series, and the summation can be obtained according to Kirchhoff's voltage law. As shown in Figure 5b, the network has 784, 100, and 10 neurons in the input layer, hidden layer, and output layer, respectively. The 8‐bit grayscale values of the input test images are encoded by the *I*_RE_ amplitudes, while the connection weights are extracted from the *I*_SE_‐modulated *R*_H_, which has 200 resistance states (Figure 5c). The highly linear modulation of *R*_H_ by the *I*_SE_ in a wide range (±100 mA) provides great advantages in MAC operations without initialization and high accuracy for neuromorphic computing. The training process was performed with 60 000 images, and another 10 000 images were used for the test process (see Section S10 for the corresponding flowchart, Supporting Information). Figure 5d shows the simulated pattern recognition accuracy as a function of training iteration. An ANN with ideal software synapses that has high linearity was used for accuracy comparison.^[^ ^29^ ^]^ Our simulation demonstrates that the ANN based on our scheme can reach a pattern recognition accuracy of ≈95%, which is close to the accuracy of ideal software‐based training (97.95%). Figure 5e,f illustrate the evolution of the SOT synaptic weights of input‐hidden and hidden‐output synaptic matrix, respectively, before and after in situ training. With the in situ training of the SOT synapses, the weights of the entire network have modified significantly. If we take account of the influence of noise in our neural network arising from the nonideal characteristics of our SOT synapses, including device‐to‐device, cycle‐to‐cycle, and reading variations (see Section S11, Supporting Information), the recognition accuracy is slightly reduced to ≈91%, indicating that the noise may have a small effect on the SOT synapse. For practical applications, a magnetic tunnel junction (MTJ) is generally used to replace the Hall bar structure, to enhance the magneto‐resistance (MR) ratio, which is helpful in increasing the recognition accuracy.

### 2.5 Power and Area Performance

In the in‐sensor memory SOT unit, the magnetic field produced by *I*_SE_ increases with the reduction of their distance and the lateral line dimensions, and also using magnetic cladding layer on the sensed current line can increase the created field on the magnet.^[^ ^30^, ^31^ ^]^ Therefore, with the optimized structure for SOT unit, the field generated by *I*_SE_ can reach typical values of several tens Oe/mA,^[^ ^31^, ^32^ ^]^ and thus maximum detectable or required current in the sensed current line could be only several hundred microampere. On the other hand, the amplitude of *I*_EN_ needed in our experiments was 30 mA with a tungsten wire of width 30 µm and thickness 5 nm. But the *I*_EN_ scales with the width of the magnet, making the IMC scheme scalable. For example, from the experimental observation, *I*_EN_ decreases from 30 to 11 mA when the width decreases from 30 to 7 µm, respectively (Section S12, Supporting Information). It should be mentioned that the device cannot be unrestricted scaled down, as the magnet should guarantee to be multidomain and domain structure continuously varies with the in‐plane field under the assistance of *I*_EN_. In a SOT‐device (or unit) with the lateral size of 0.6 × 1 µm^2^, which exhibits a good linear relationship between *R*_H_ and *H*_x_, the current amplitude is reduced to 1.2 mA (Section S12, Supporting Information). The current amplitude can be further decreased by reducing the thickness of the wire.

In the metallic SOT units, the dominant mechanism for power dissipation is Joule heating (*I* ^2^ *R* loss). The resistivity of the SOT device is 186 µΩ cm, as measured in our experiments and similar to the values reported by Zhang et al.^[^ ^33^ ^]^ As discussed above, the Joule heating mostly caused by *I*_EN_ was experimentally estimated to be ≈552 µW without optimization for the device with the lateral size of 0.6 × 1 µm^2^. Accordingly, our in‐memory analog adder with two input currents and multiplier consumes ≈1.7 mW (552 µW × 3) and 552 µW, respectively, which are comparable to the analog implementation of arithmetic operations by the state‐of‐the‐art CMOS‐based technologies (2.7 mW for adder using 0.25 µm technology node and 232 µW for multiplier using 0.18 µm node). In contrast, a reduction in area overhead of more than one to two orders of magnitude is obtained for the demonstrated scheme compared with CMOS‐based technologies. The details of the calculation as well as comparative charts can be found in Section S13 (Supporting Information). Moreover, the enable current and consequently power dissipation could be further lowered by using small anisotropy field magnet or/and other heavy metals with larger spin Hall angles, such as CuIr.^[^ ^34^ ^]^

## 3 Conclusion

To summarize, we have reported a spin‐based IMAC scheme, which provides the area and energy efficient strategy for on‐chip mathematical computation, signal and image processing, and neuromorphic computing. Besides, the divider could be implemented using our scheme with the help of operational amplifier.^[^ ^35^ ^]^ Our proposed architecture of in‐sensor electrical current computing is also possible to real‐time monitor the currents in the conductive paths and nodes for the power management of integrated circuits. For practical applications, MTJs would be utilized to replace the Hall bar structures, consequently, the noncontact current is sensed and memorized in the free layer of MTJ. Therefore, present proposed in‐memory sensing unit would evolve into the magnetic field‐assist SOT magnetic random access memory (SOT‐MRAM) cell structure (Section S14, Supporting Information). It indicates our proposed IMAC architecture can take advantage of the developed MRAM technologies, such as optimizing individual SOT unit and their interconnected structure to lower area overhead.

## 4 Experimental Section

### Sample Preparation

Magnetron sputtering process was used to deposit a film structure of W (5 nm)/CoFeB (1.1 nm)/MgO (2 nm)/Ta (2 nm) on a thermally oxidized Si substrate at room temperature. Then the fabrication of the devices was carried out as the following descriptions.

Step 1: photolithography and etching. The thin‐film stack was fabricated into Hall bars (so‐called SOT devices in the main text) by photolithography (using a deep ultraviolet lithography machine) and argon‐ion milling (using MIBE 150A etching machine) after the cleaning procedure. The dimension of the core area for the SOT device is 30 × 30 µm^2^. Step 2: Al_2_O_3_ film deposition. Atomic layer deposition (ALD) was used to deposit 50 nm Al_2_O_3_ on the prepared SOT devices array as the insulation layer. Step 3: expose the electrodes of the SOT devices. First, a photolithography was done to expose the areas of all the electrodes, and the other areas were covered with photoresist. Next, the argon‐ion milling (MIBE 150A) was used to etch the Al_2_O_3_ about 40 nm. Then, the buffer oxide etch (BOE) was utilized to etch the remaining Al_2_O_3_ (about 10 nm). Finally, the photoresist was removed. Step 4: deposit Au. In order to fabricate the Au paths that can generate a planar magnetic field and cap on electrodes with Au at the same time, electron beam evaporation (EBE) was used to grow a Ti (10 nm)/Au (60 nm) bilayer. The width of the channel in the Au paths were 50 µm, and the dimension of the device electrodes is 100 × 100 µm^2^.

### Electrical Characterization

For the AHR measurements, a d.c. current source (Keithley model 6221) was used to apply currents, a nanovoltmeter (Keithley model 2182A) to measure the Hall voltage, and Keithley model 2400 (or 2410) to apply currents in the Au paths.

### MOKE Imaging

MOKE images were used to magnetically image the domain nucleation dominated switching mode in the CoFeB layer. After saturating the magnet in the +z direction, then an image was taken to serve as the reference image. Different external magnetic field *H*_x_ under a writing current of 30 mA for 0.5 s was next applied, another image was taken. The first reference image was subtracted from the second image to get the final MOKE image.

## Conflict of Interest

The authors declare no conflict of interest.

## Author Contributions

R.L., M.S., Z.G., and S.L. contributed equally to this work. L.Y. conceived the project and designed the experiments. R.L., S.L., and Y.T. fabricated the samples. R.L. and S.L. implemented the experimental set‐up and measurements. R.L. and S.Z. performed the MOKE measurements. M.S., Z.C., W.D., and L.Y. performed the simulations. R.L., L.Y., M.S., Z.G., and S.Z. analyzed the results. M.S., X.Y., Y.W., J.H., Y.C., Z.Y., and W.T. provided the theoretical support. L.Y., R.L., Z.G., and M.S. wrote the manuscript. All authors discussed the data and contributed to the manuscript.

## Supporting information

Supporting Information

## Data Availability

The data that support the findings of this study are available from the corresponding author upon reasonable request.
