# Supplementary material for: In‐Memory Mathematical Operations with Spin‐Orbit Torque Devices
Source: Adv Sci (Weinh). 2022 Jul 10;9(25):2202478. doi: 10.1002/advs.202202478 (PMC9443454; doi:10.1002/advs.202202478)
Supplement: Supplementary file 1 — Supporting Information [file ADVS-9-2202478-s001.pdf]

ADVANCED SCIENCE
Open Access

Supporting Information

for *Adv. Sci.*, DOI 10.1002/advs.202202478

In-Memory Mathematical Operations with Spin-Orbit Torque Devices

*Ruofan Li, Min Song, Zhe Guo, Shihao Li, Wei Duan, Shuai Zhang, Yufeng Tian, Zhenjiang Chen, Yi Bao, Jinsong Cui, Yan Xu, Yaoyuan Wang, Wei Tong, Zhe Yuan, Yan Cui, Li Xi, Dan Feng, Xiaofei Yang, Xuecheng Zou, Jeongmin Hong and Long You**

Supplementary Information for

In-memory mathematical operations with spin-orbit torque devices

Ruofan Li[#], Min Song[#], Zhe Guo[#], Shihao Li[#], Wei Duan, Shuai Zhang, Yufeng Tian, Zhenjiang Chen, Yi Bao, Jinsong Cui, Yan Xu, Yaoyuan Wang, Wei Tong, Zhe Yuan, Yan Cui, Li Xi, Dan Feng, Xiaofei Yang, Xuecheng Zou, Jeongmin Hong, Long You[*]

[#] *These authors contributed equally to this work.*

[*] E-mail: lyou@hust.edu.cn

Supplementary Text

Supplementary Section 1. Magnetic transport characterizations of the SOT device.

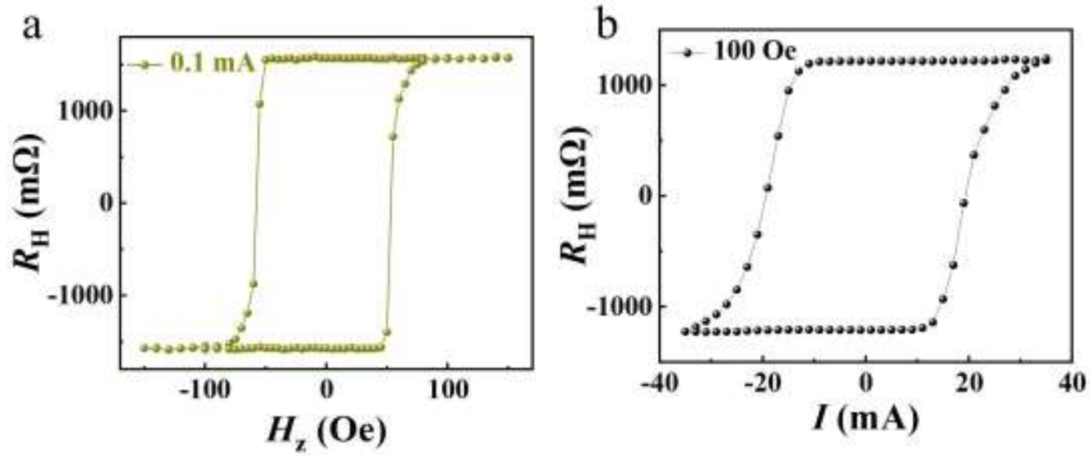

Fig. S1. a, Magnetic field along *z* direction (H_z) induced magnetization switching of the SOT device. **b,** Current induced magnetization switching of the SOT device with H_x=100 Oe.

Supplementary Section 2. COMSOL simulations on the generated magnetic field by I_{SE} flowing in the Au path.

We use the COMSOL simulation software to verify that the generated magnetic field by I_{SE} is just along the *x* direction in the SOT device, as schematically shown in Fig. 1d in the main text.

In the simulation model (Fig. S2a), we set the Au path with a width of 50 µm, length of 600 µm and height of 60 nm, as in the actual experiments. An I_{SE} current in *y* direction with a density of 3.34 × 10^{10} A/m^2 was applied in the Au path. Fig. S2 shows the simulated magnetic field distribution results in

the *xz* plane ($y = 0$).

The distribution of *x*-component (H_x) of the generated magnetic field is shown in Fig. S2b. At a given height (i.e. *z* coordinate value), it can be seen a uniform H_x is generated in the locations sitting immediately under or above the Au path, within the *x* range of [-15 μm, 15 μm]. In contrast, H_y and H_z almost keep zero in corresponding locations as shown in Fig. S2c and Fig. S2d.

This phenomenon can be understood by the Ampere's law. Generally, when electrical current is injected into a wire, the circular concentric magnetic fields will be induced around the wire. However, in our case the Au path has a width of 50 μm and thickness of 60 nm, which should be considered as a plane conductor relative to the Hall bar structure underneath the Au path, not a line. Such a case can be considered as an array of infinitesimally thin wire conductors arranged on a flat plane.[1] The immediate magnetic field lines to the wires may keep their closed loop nature. But the induced fields far away from the wires will join each other and result in the straight line-like path, i.e. the constant H_x in the Hall bar structure.

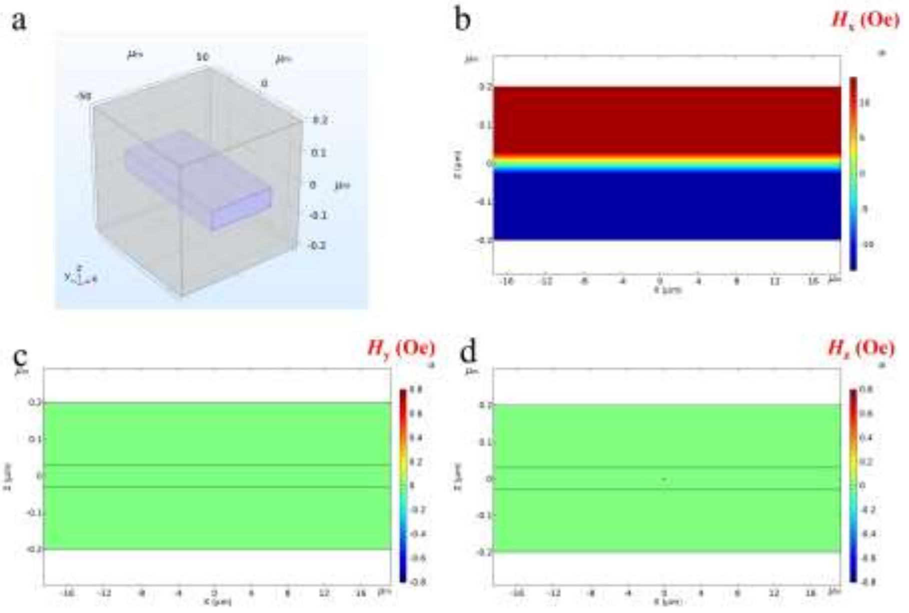

Fig. S2. a, The model structure for the COMSOL simulation. The distribution of **b,** *x*- **c,** *y*- **d,** *z*-component of the generated magnetic field in the *xz* plane ($y = 0$).

Supplementary Section 3. The estimation of I_{SE}-generated in-plane magnetic field at the SOT device.

We firstly measured R_H-H_x curve at I_{EN} = 30 mA with H_x scanning forward and backward between −12 and +12 Oe. As shown in Figure S3, the experimental results show the R_H varies linearly with H_x.

Then we used I_{SE}, which could generate magnetic field at the SOT device, to replace the role of H_x. From the R_H-I_{SE} curve (Fig. S3), it can be found that R_H also varies linearly with I_{SE}. Typically, when I_{SE} changes between −100 and +100 mA, the R_H variation induced by I_{SE} exactly equals to the one caused by H_x, indicating the correspondence between I_{SE} and H_x , i.e. I_{SE} ranging from −100 to 100 mA corresponding to H_x from −12 to +12 Oe, respectively.

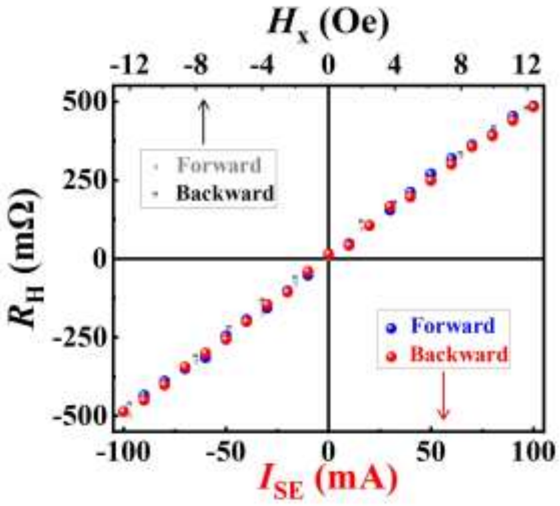

Fig. S3. R_H as a function of H_x and I_{SE}, respectively.

Supplementary Section 4. The investigation of *k* variation under different I_{EN} in real-time or offline sense case.

Corresponding to collecting R_H during I_{EN} is switched on or applying a small reading current I_{RE} (here 0.1 mA) after switching off I_{EN}, the SOT device can real-time and offline sense I_{SE} through R_H, respectively. However, k is different in the two cases. Exactly speaking, k in real-time sense case is lower than that in offline case. The reason could be explained as follows. In the real-time sense case, the Joule heating caused by I_{EN} may decrease the anomalous Hall coefficient and/or magnetization, and thus leads to a reduced R_H at an arbitrary given I_{SE} (or reduced k). However, the heating effect can be neglected in the offline sense case as the reading process is performed using a small reading current of 0.1 mA. Fig. S4 shows the comparison of the coefficient k in real-time and offline sense cases under different I_{EN}. With the increase of I_{EN}, the k difference in two cases become more distinct, since the larger I_{EN} leads to a higher temperature in the real-time sense case.

Moreover, we investigated k variations under different I_{EN} in the real-time sense case or offline sense case and illustrated the results in Fig. S5. With I_{EN} changing from 30 to 33 mA, k slightly increases in both real-time and offline cases. This can be attributed to more grains reversal caused by

the increase of I_{EN}. With increase of I_{EN} from 33 to 37 mA, k remains almost constant for offline sense case. Whereas, k starts to decrease for real-time sense case due to heating-induced R_H reduction[2]. For further increasing k from 37 mA, k will decrease in both cases, which could be explained by the heating-induced demagnetization of the SOT device [3].

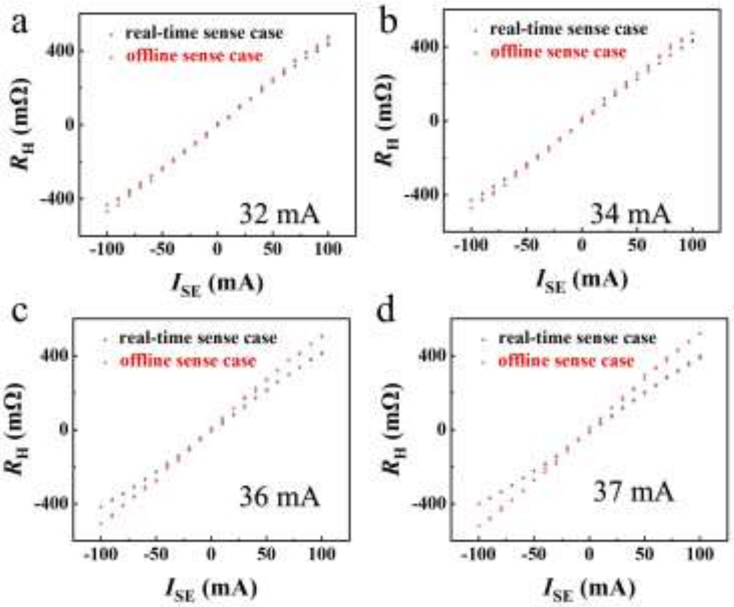

Fig. S4. The comparison of the coefficient k in real-time and off-line sense cases under **a,** I_{EN} = 32 mA, **b,** I_{EN} = 34 mA, **c,** I_{EN} = 36 mA, and **d,** I_{EN} = 37 mA.

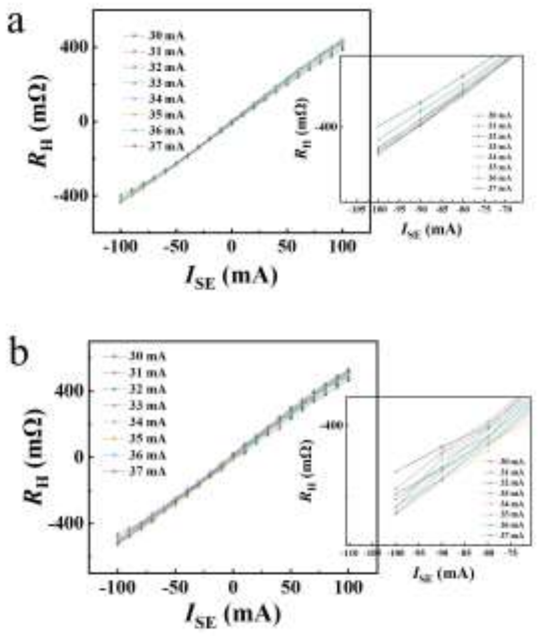

Fig. S5. The coefficient k variations **a,** in real-time sense case, and **b,** in offline sense case, respectively.

Supplementary Section 5. The performance of two-input adder with different input current combinations.

Fig. S6a shows the R_H-I_{SE} curve of each SOT unit ($R_{H(in1)}$, $R_{H(in2)}$ and $R_{H(out)}$) in the two-input configuration. It can be seen the three SOT units have good consistency, which indicates they have the same coefficient *k*. In order to confirm the stability of our configuration, we applied a series of I_{in2} (0 mA, –20 mA, –40 mA) for measurements. As expected ($R_{H(out)} = R_{H(in1)} + R_{H(in2)} = k \times I_{in1} + R_{H(in2)}$ in the main text), the collected R_H values also show good summation relationships, as shown in the Fig. S6b, c and d. Noting that, as the linear range for I_{SE} is from –100 to 100 mA, the applied I_{in1} in the experiments is limited at a given I_{in2} to ensure I_{out} is in the linear range. For instance, under I_{in2}= –20 mA, we applied I_{in1} varying from –80 to 80 mA.

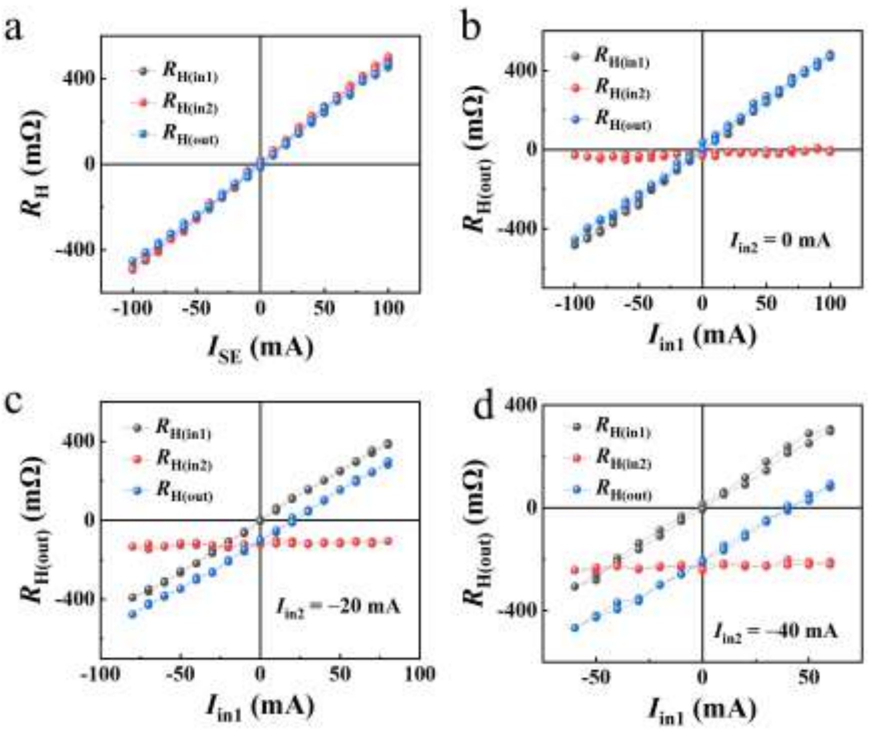

Fig. S6. a, The R_H-I_{SE} curves for each SOT unit. R_H as a function of input current I_{in1} under **b,** I_{in2}= 0 mA, **c,** I_{in2}= –20 mA and **d,** I_{in2}= –40 mA.

Supplementary Section 6. The performance of three-input adder with different input current combinations.

Fig. S7a shows the R_H-I_{SE} curve of each SOT unit ($R_{H(in1)}$, $R_{H(in2)}$, R_H', $R_{H(in3)}$ and $R_{H(out)}$) in the three-input configuration, indicating the same coefficient *k*. Similarly, we also applied a series of different combinations of (I_{in1}, I_{in2}) for measurements, that are (20 mA, 30 mA), (–10 mA, 30 mA), (10

mA, –10 mA) and (–20 mA, –30 mA). As expected ($R_{H(out)} = R_{H(in1)} + R_{H(in2)} + R_{H(in3)} = R_H' + k \times I_{in3}$ in the main text), the collected R_H values also show good summation relationships, as shown in the Fig. S7b, c, d and e.

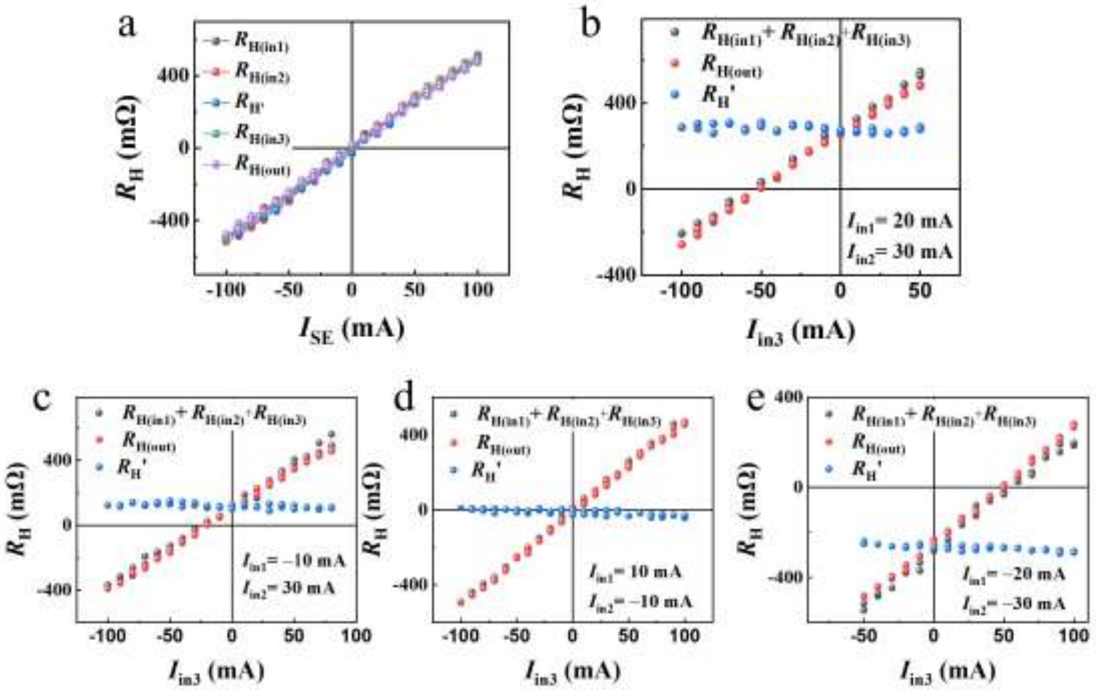

Fig. S7. a, The R_H-I_{SE} curves for each SOT unit. R_H as a function of input current I_{in3} with different combinations of **b,** I_{in1}= 20 mA, I_{in2}= 30 mA, **c,** I_{in1}= –10 mA, I_{in2}= 30 mA, **d,** I_{in1}= 10 mA, I_{in2}= –10 mA, **e,** I_{in1}= –20 mA, I_{in2}= –30 mA.

Supplementary Section 7. Edge detection implementation based on our scheme.

Edge detection is a basic operation used for feature detection or extraction in image processing, which aims to identify points in an image where brightness of image change sharply and find discontinuities[4]. The purpose of edge detection is to reduce the amount of useless data in an image and preserves the structural properties for further image processing.

Various edge detection techniques have been proposed, among which the gradient-based one is performed by taking the first order derivative of the image[5]. First-order derivatives in image processing are implemented using the magnitude of the gradient. For a function f(x, y), the differential at coordinates (x, y) is denoted as the two dimensional column vector

$$\nabla \mathrm{f} = \mathrm{G}[f(x, y)] = \begin{bmatrix} G_x \\ G_y \end{bmatrix} = \begin{bmatrix} \frac{\partial f}{\partial x} \\ \frac{\partial f}{\partial y} \end{bmatrix} \qquad (1)$$

The quantity ∇f is known as the gradient of a vector. The magnitude of vector, denoted as M(x, y), which represents the edge strength:

$$M(x,y)= \text{magnitude } (\nabla f)=|G| = \sqrt{G_x^2 + G_y^2} \qquad (2)$$

where, G_x and G_y are the gradients in x and y direction, respectively.

To simplify the computation, this quantity can be approximated by using absolute values, i.e.

$$M(x,y) \approx |G_x| + |G_y| \qquad (3)$$

As a simple and quick method to calculate the 2D gradient magnitude, the Roberts operator with a pair of 2 × 2 convolution kernels is utilized. The Roberts cross operator provides a simple proximity of 2 × 2 mask:

G_x=

1	0
0	-1

G_y=

0	1
-1	0

Considering a 2 × 2 grayscale values array as follows, where $(x,)$ denotes the grayscale values in location (x,y):

f(x,y)	f(x,y+1)
f(x+1,y)	f(x+1,y+1)

Then, eq. (3) can be further written as given below[6]:

$$\nabla f = |f(x,y) - f(x+1,y+1)| + |f(x,y+1) - f(x+1,y)| \qquad (4)$$

For the hardware implementation of such computing, we propose a scheme as shown in Fig. S8. Firstly, a circuit is needed to compare the magnitudes of grayscale values in the diagonal direction and assign the larger value as positive inputs and smaller one as negative (part I in Fig. S8). Then two analogue subtractors are adopted with part I in Fig. S8 to implement the absolute values of the differences between diagonally adjacent pixels in the surrounding 2 × 2 greyscale matrix. The outputs of subtractors are finally fed into an analogue adder to obtain the final gradient magnitude. A flowchart to describe the whole process for edge detection are shown in Fig. S9.

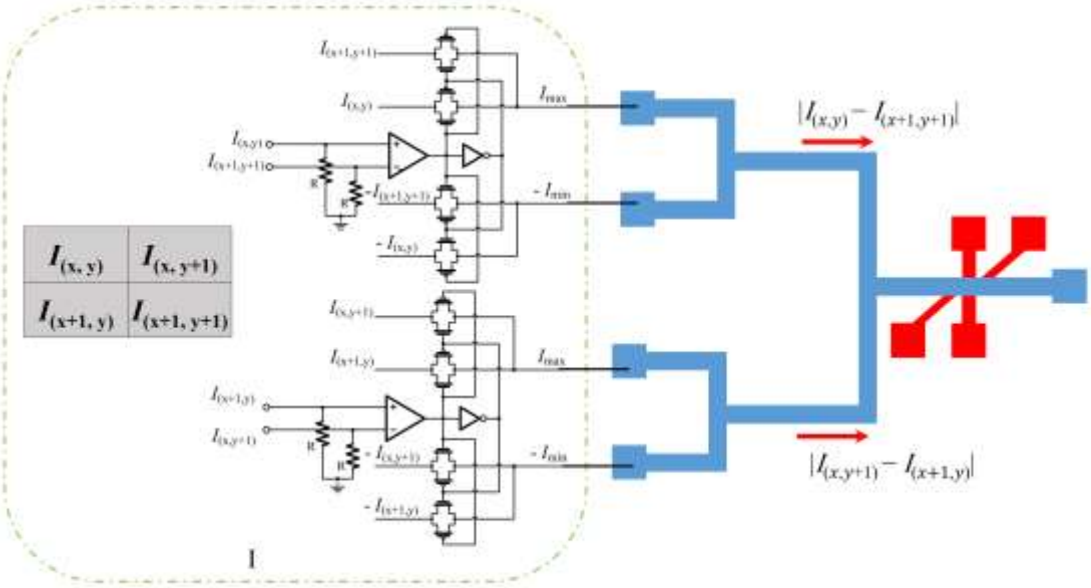

Fig. S8. Edge detection hardware implement proposal using Roberts' algorithm.

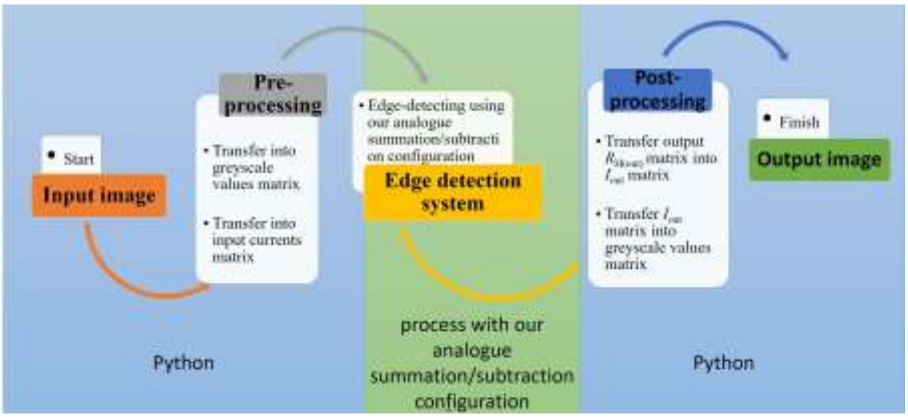

Fig. S9. Edge detection flowchart in our work.

Supplementary Section 8. Pixel differences between the ideal output and the output based on our scheme.

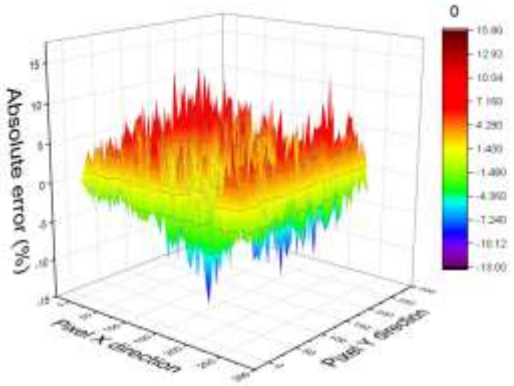

Fig. S10. Pixel differences between the ideal output and the output based on our scheme.

Supplementary Section 9. The range of I_{RE} for multiplication computing in the offline sense case.

In order to remain k (k = R_H/I_{SE}) constant with independence of I_{RE} for the multiplication computing, the I_{RE} should have negligible effect on R_H. We firstly applied a fixed I_{SE} (100 mA) with I_{EN} = 30 mA to write R_H, and then read U_H with a varying I_{RE} to obtain R_H. The black line in Fig. S11a shows that the R_H remains constant with I_{RE} varying from -10 to 10 mA and then back to –10 mA. With applying different I_{SE} to write R_H, the I_{RE} in the range of [–10 mA, 10 mA] also has negligible effect on R_H. Fig. S11b shows the R_H as a function of I_{SE} with different I_{RE} (±0.1, ±2, ±4, ±6, ±8, ±10 mA). It can be found that these curves are almost identical and have the same slope k, which confirms that the I_{RE} in such range will not alter the k.

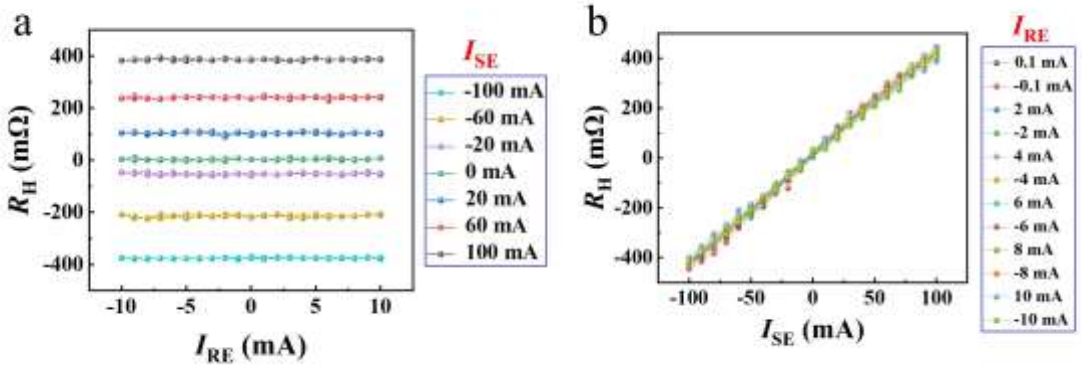

Fig. S11. The range I_{RE} of for multiplication computing. **a,** R_H-I_{RE} curves with I_{RE} scanning forward and backward between –10 mA and 10 mA, under different I_{SE}. **b,** R_H-I_{SE} curves with I_{SE} scanning forward and backward between –100 mA and 100 mA, under different I_{RE}.

Supplementary Section 10. The flowchart of the training process.

The in-situ training method used in the main text (Fig. 5) consists of two stages: feed-forward inference and error back-propagation. When the training starts, weights of each layer are initialized with He Initialization, which is suitable for the Rectified Linear Unit (ReLU) activation function[7]. Then, an image is randomly selected from the training dataset and converted into a set of current vectors which are applied to the array pixel by pixel. The output vectors of the hidden layer are activated by the neurons and fed into the output layer to get the final result.

The training process enters the back-propagation stage when all the inferences of a batch of images are completed. In this work, we used the cross-entropy error loss function (equation) as the goal of the training process, which needs to be minimized by tuning the network weights.

$$E = -\sum_{i=1}^{n} t_i * \ln y_i \quad (0 \le y_i \le 1, \sum_{i=1}^{n} y_i = 1)$$

where n is the total number of the classes, and $t_1 \sim t_n$ are the one-hot label of the input image, y_i is the predicted possibility of class i from the network.

The gradient of each synapse is calculated through the error back-propagation algorithm in software. After that, the Stochastic Gradient Descent (SGD) algorithm is applied to update the weights along the direction of steepest descent for the loss function. The desired weight update equation is:

$$\Delta w_{new} = \left[(w_{old} + \Delta Read) - \eta \frac{\partial E}{\partial w_{old}}\right] \cdot (1 + \Delta C_2 C) \cdot (1 + \Delta D_2 D)$$

Where η is the optimized learning rate, $\frac{\partial E}{\partial w_{old}}$ is the gradient of the weight. $\Delta Read$, $\Delta C_2 C$ and $\Delta D_2 D$ represent the variations from non-ideal characteristics of reading, cycle-to-cycle and device-to-device, respectively.

When it comes to the last batch of one epoch, an iteration is completed and the next one will start. The training process will stop if the accuracy of the network reaches an acceptable range.

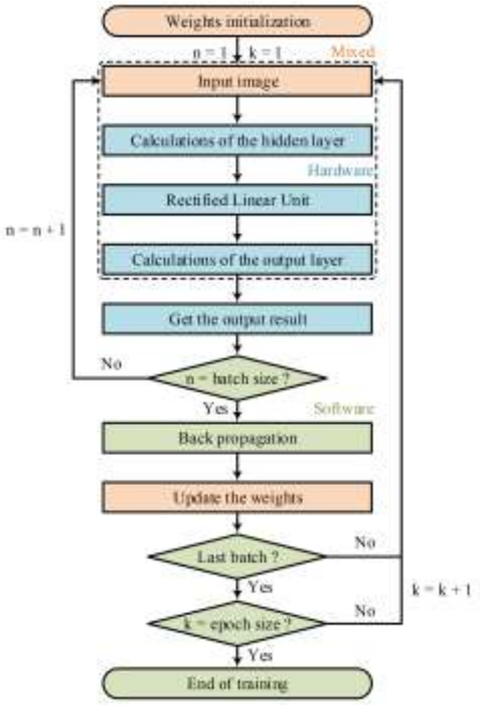

Fig. S12. Flowchart of the in-situ training. The blue boxes show the steps implemented in hardware, while those in green boxes were implemented in software. In addition, the processes that require both hardware and software were indicated in orange.

Supplementary Section 11. The variations of our synapses.

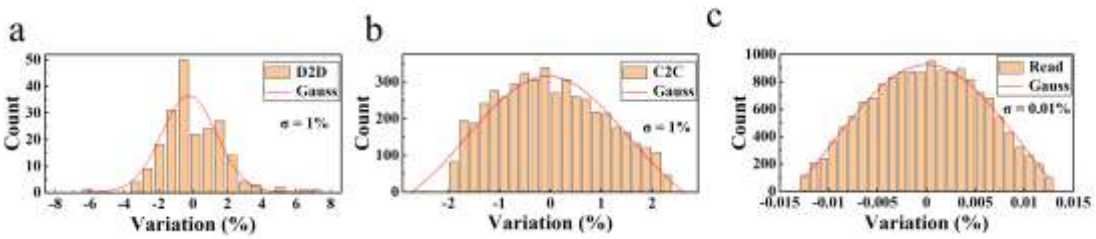

Fig. S13. The variations in our neural network from non-ideal characteristics of our synapses including **a,** device-to-device, **b,** cycle-to-cycle and **c,** reading variations.

Supplementary Section 12. The scaling performance of the SOT device.

Fig. S14a shows R_H as a function of H_x for the SOT device with the dimension of 7 μm × 7 μm, under I_{EN} = 11 mA. The R_H has a continuous and linear response with H_x (i.e. I_{SE}) under the application of I_{EN}. Moreover, when the device dimension is reduced to 0.6 μm × 1 μm, which is fabricated by electron beam lithography (EBL) and ion milling, a good linear relationship between R_H and H_x can still be observed, as shown in Fig. S14b. Noting that, the required I_{EN} is reduced to 1.2 mA.

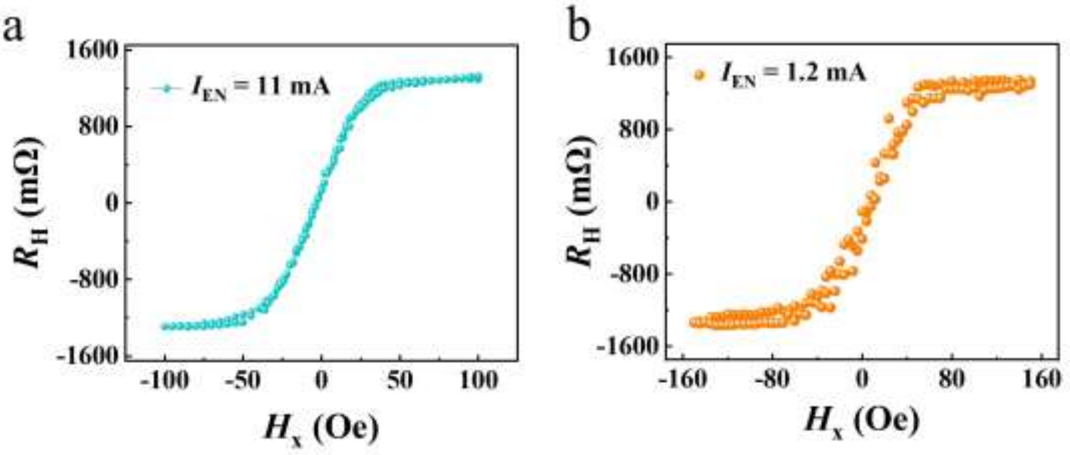

Fig. S14. The R_H-H_X curve under for **a,** 7 μm × 7 μm and **b,** 0.6 μm × 1 μm SOT device.

Supplementary Section 13. The energy/area estimation and comparison with CMOS-based analogue computing circuits.

We calculate the energy dissipation and area overhead using the scaled device with the feature size of 0.6 μm × 1 μm. In our scheme, most of the energy dissipation occurs in the form of Joule heating, which is mostly contributed by I_{EN}. As demonstrated in the above section, the I_{EN} for the scaled device is 1.2 mA. The resistivity of the heterostructure is 186 μΩ·cm, as measured in our experiments. Thus,

the energy dissipation for one unit is approximately 552 μW. Accordingly, the energy dissipation for the two-input adder which consists of three SOT units is 3 × 552 μW, i.e., about 1.7 mW, and the multiplier that consume a single unit is 552 μW. Fig. S15a and 15b show the layout of single unit and two-input adder, respectively, where the minimum width and spacing of various elements is *F* (*F* denotes the feature size), *W* and *L* represents width and length of magnets, respectively. Here, we use the 0.18 μm technology node for area overhead estimation, which is widely used for analogue integrated circuits fabrication. As an example, when the size changes from 7 μm × 7 μm to 0.6 μm × 1 μm device, the area overhead for a single unit decreases from 59.6 μm^2 to 2.27 μm^2. Meanwhile, the estimated area overhead for two-input adder is 243.98 and 10.21 μm^2, respectively.

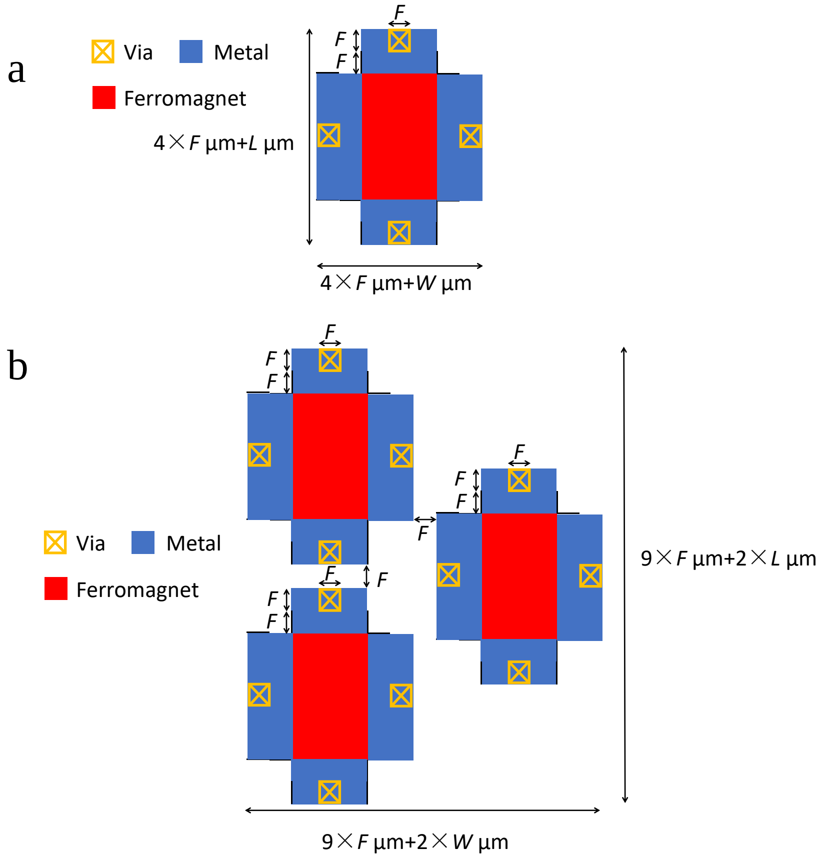

Fig. S15. The layout schematic of (a) a single unit and (b) two-input adder for area overhead estimation.

Table S1 and S2 compares key data of our two-input analogue adder (here named SOT adder) and multiplier (here named SOT multiplier) with their CMOS counterparts, respectively. For both adder and multiplier, our schemes consume the minimal number of basic elements. Compared to the CMOS multipliers using currents as inputs, the SOT multiplier has the maximal input range. In terms of power consumption, our scheme (both adder and multiplier) is comparable to the CMOS technology, while a

reduction in area overhead of more than one to two orders is obtained compared with the CMOS counterparts. In addition, our SOT adder can store the summation results while performing the computing. This saves a lot of energy compared to the CMOS adders based on Von Neumann architecture.

Table S1

Reference	Technology	Number of elements	Input range	Active area (μm2)	Power consumption	Memorizing function?
Ref. 8	2 μm	8[a]	±0.8 V	>3152	NA	NO
Ref. 9	6.5 μm	6[a]	±3 V	135 x 145	0.5 mW	NO
Ref. 10	1.2 μm	10[a]	±2 V	95.4 x 455.4	NA	NO
Ref. 11	0.25μm	6[a]	±0.4 V	390	2.7 mW	NO
This work[#]		3[b]	±50 mA	10.2	1.7 mW	YES

[a]The number of transistors
[b]The number of SOT-units
[#]Calculated using the parameters of the 600 nm-width SOT device

Table S2

Reference	Technology	Number of elements	Input range	Active area (μm^2)	Power consumption (μW)	Quadrant
Ref. 12	0.18 μm	36[a]	±100 mV	NA	588	four
Ref. 13	0.18 μm	17[a]	NA	600	60	two
Ref. 14	0.18 μm	28[a]	±20 μA	147	700	four
Ref. 15	0.35μm	12[a]	±10 μA	594	232	four
This work[#1]		1[b]	±100 mA (I_{SE}); ±120 μA (I_{RE})	2.3	552	four

[a]The number of transistors
[b]The number of SOT-units
[#]Calculated using the parameters of the 600 nm-width SOT device

Supplementary Section 14. The basic in-memory sensing unit and corresponding adder configuration with MTJ structure.

For the actual applications in the future, the Hall bar structure can be replaced by the MTJ, which is schematically shown in Fig. S16. As mentioned in the main text, our proposed in-memory sensing unit can be regarded as the combination of field-switched MRAM and SOT-MRAM, i.e., magnetic

field-assist SOT-MRAM. Our unit adds a wire, based on the SOT-MRAM, in which I_{SE} can be applied to generate magnetic field for detecting. In such configuration, the basic unit has 5 terminals in total.

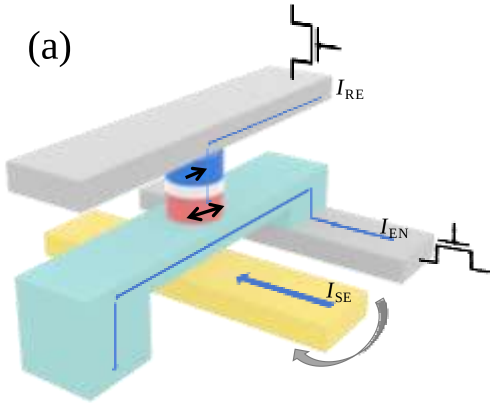

Fig. S16. The schematic of the spatial structure of the basic in-memory sensing unit using the MTJ and corresponding two-input adder.

Fig. S17 shows the schematic of our unit with two accessing transistors and corresponding biasing conditions for writing and reading operations, which is similar to the SOT-MRAM cell[16]. For writing operation, the Write Word-Line (WWL) is assigned as V_{DD} to turn on the access transistor. The Write Bit-Line (WBL) is assigned as V_{EN} to trigger I_{EN}, with Source line (SL) connected to the ground. Meanwhile, I_{SE} is applied in the Sensing Curren Line (SCL) during the application of V_{EN}. For the reading process, the Read Word-Line (RWL) is assigned as V_{DD} to switch on the other access transistor. Then the reading current (I_{RE}) is applied in the Read Bit-Line (RBL) to trigger the reading voltage, which can be detected by the sense amplifier.

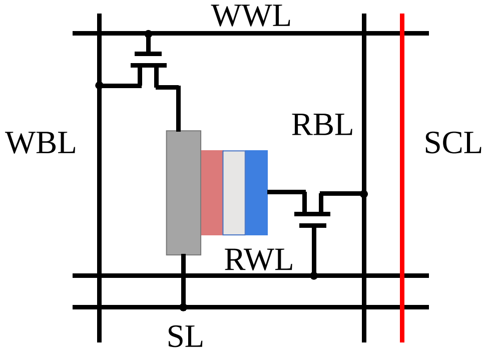

	Write	Read
WWL	V_{DD}	0
RWL	0	V_{DD}
RBL	0	I_{RE}
WBL	V_{EN}	0
SCL	I_{SE}	/
SL	0	0

Fig. S17 The schematic of our basic in-memory sensing unit based on the SOT-MRAM cell and corresponding biasing conditions for summation and reading operations.

References

1. Sedki M. Riad, P.E.Iman M. Salama, Electromagnetic Fields and Waves: Fundamentals of Engineering, 2020 McGraw-Hill Education
2. Hao, Q. & Xiao, G. Giant spin Hall effect and magnetotransport in a Ta/CoFeB/MgO layered structure: A temperature dependence study. *Phys. Rev. B.* **91,** 224413 (2015).
3. Huang, K. F., Wang, D. S., Tsai, M. H., Lin, H. H. & Lai, C. H. Initialization-Free Multilevel States Driven by Spin–Orbit Torque Switching. *Adv. Mater.* **29,** 1601575 (2017).
4. Rashmi, R., Kumar, M. & Saxena, R. Algorithm and Technique on Various Edge Detection: A Survey. *Signal & Image Processing,* An International journal (SIPU) **4,** (2013).
5. Spont´on, H. & Cardelino, J. A Review of Classic Edge Detectors, *Image Processing On Line,* **5,** 90–123 (2015).
6. Gupta, S., Gupta, C. & Chakarvarti, S. K. Image Edge Detection: A Review. *International Journal of Advanced Research in Computer Engineering & Technology (IJARCET)* **2,** (2013).
7. He, K., Zhang, X., Ren, S. & Sun, J. Delving Deep into Rectifiers: Surpassing Human-Level Performance on ImageNet Classification. *Proceedings of the IEEE International Conference on Computer Vision (ICCV),* 1026-1034 (2015).
8. Diaz-Sanchez, A. & Ramirez-Angulo, J. A compact high frequency VLSI differential analog adder. *Proceedings of the 39th Midwest Symposium on Circuits and Systems* **1,** 21-24 (1996).
9. Al-Nsour, M. & Abdel-Aty-Zohdy, H. S. Simple low power analogue MOS voltage adder. *Electro. lett.* **35,** 552 – 553 (1999).
10. Xu, J., Siferd, R. & Ewing, R. L. High Performance CMOS Analog Arithmetic Circuits. *Analog Integrated Circuits and Signal Processing* **20,** 193–201 (1999).
11. Minaei, S., Göknar, I. C., Yıldız, M. & Yuce, E. Memstor, memstance simulations via a versatile 4-port built with new adder and subtractor circuits. *International Journal of Electronics,* **102,** 911-931 (2015).
12. Tanno, K., Ishizuka, O. & Tang, Z. Four-quadrant CMOS current-mode multiplier independent of device parameters. *IEEE Transactions on Circuits and Systems II: Analog and Digital Signal Processing,* **47,** 473-477 (2000).
13. Popa, C. Improved Accuracy Current-Mode Multiplier Circuits With Applications in Analog Signal Processing. *IEEE Transactions on Very Large Scale Integration (VLSI) Systems,* **22,** 443-447 (2014).
14. Beyraghi, N. & Khoei, A. CMOS design of a low power and high precision four-quadrant analog multiplier. *AEU - International Journal of Electronics and Communications,* **69,** 400-407 (2015).
15. Al-Absi, M. A. & As-Sabban, I. A. A New Highly Accurate CMOS Current-Mode Four-Quadrant Multiplier. *Arab J. Sci. Eng.* **40,** 551–558 (2015).
16. Z. He, Y. Zhang, S. Angizi, B. Gong, D. Fan, Exploring a SOT-MRAM Based In-Memory Computing for Data Processing, *IEEE Transactions on multi-scale computing systems,* **2018**, 4, 676-685.
